# A combinatorial approach towards the design of nanofibrous scaffolds for chondrogenesis

**DOI:** 10.1038/srep14804

**Published:** 2015-10-07

**Authors:** Maqsood Ahmed, Tiago André da Silva Ramos, Febriyani Damanik, Bach Quang Le, Paul Wieringa, Martin Bennink, Clemens van Blitterswijk, Jan de Boer, Lorenzo Moroni

**Affiliations:** 1 University of Twente, Department of Tissue Regeneration, Enschede, 7500 AE, The Netherlands; 2 University of Twente, Department of Nanobiophysics, Enschede, 7500 AE, The Netherlands; 3 Faculty of Engineering, University of Oporto, 4200-465 Porto, Portugal

## Abstract

The extracellular matrix (ECM) is a three-dimensional (3D) structure composed of proteinaceous fibres that provide physical and biological cues to direct cell behaviour. Here, we build a library of hybrid collagen-polymer fibrous scaffolds with nanoscale dimensions and screen them for their ability to grow chondrocytes for cartilage repair. Poly(lactic acid) and poly (lactic-co-glycolic acid) at two different monomer ratios (85:15 and 50:50) were incrementally blended with collagen. Physical properties (wettability and stiffness) of the scaffolds were characterized and related to biological performance (proliferation, ECM production, and gene expression) and structure-function relationships were developed. We found that soft scaffolds with an intermediate wettability composed of the highly biodegradable PLGA50:50 and collagen, in two ratios (40:60 and 60:40), were optimal for chondrogenic differentiation of ATDC5 cells as determined by increased ECM production and enhanced cartilage specific gene expression. Long-term cultures indicated a stable phenotype with minimal de-differentiation or hypertrophy. The combinatorial methodology applied herein is a promising approach for the design and development of scaffolds for regenerative medicine.

In the natural tissue microenvironment, the extracellular matrix (ECM) provides an elaborate network of chemical, physical, and mechanical cues that influence cellular behaviour^1^,^2^. With a view to recapturing these features, the development of 3D scaffolds as templates for regeneration has formed the basis of tissue engineering. Such scaffolds have already found clinical use in preliminary proof-of-principle studies; however, widespread success remains dependent on understanding the basic cell-scaffold interactions^3^,^4^.

Cell behaviour can be quite diverse in response to a scaffold. Features such as surface chemistry, morphology and mechanics, can have an influence over cell fate. Benoit *et al.* demonstrated that phosphate groups tethered to poly(ethylene) glycol (PEG) hydrogels, for example, induce osteogenic differentiation of human mesenchymal stromal cells (hMSCs) whilst hydrophobic tertiary butyl groups promote adipogenesis^5^. Simply by altering the small molecule functional groups present in the hydrogel, the authors were able to direct cell fate providing a simple means by which to control complex biological processes. Meanwhile, bulk stiffness has also been shown to direct hMSC lineage specification with soft matrices promoting neurogenesis, stiffer matrices comparable to muscle were myogenic and comparatively rigid matrices proving to be osteogenic^6^. More recently, this has been challenged by Trappmann *et al.* who provide data which suggests that rather than bulk stiffness, it is the surface porosity that is the lineage determining feature of hydrogels^7^. Topographical features such as porosity can influence a number of cellular processes^8^. Highly disordered arrays of pores resulted in increased bone mineral production and the osteogenic differentiation of hMSCs even in the absence of chemical supplements^9^. Thus, cellular responses to biomaterials are complex, multifactorial and interdependent. This is further exacerbated as tissues are multicellular structures that signal to each other through a range of dynamic cell-cell and cell-matrix interactions. Understanding this complex interplay of signals present in the cellular niche is critical to the successful translation of any tissue engineering and regenerative medicine product.

Traditional biomaterials research has historically been driven by a “trial and error” approach, focused on preparing samples one at a time for characterization and testing. However, with a multitude of parameters to test, ranging from variations in material properties to cell response that must be measured and evaluated, progress has been limited. Furthermore, the lengthy and costly evaluation process places restrictions on the number of cell types, materials and external stimuli that can be examined. Therefore, we and others have pioneered the use of combinatorial and high-throughput methodologies offering the possibility to screen the cellular response to a large number of scaffold properties in one experiment^10^,^11^,^12^,^13^.

Using a combination of mathematical algorithms and microfabrication techniques, we have previously developed a platform to examine the effect of over 2000 topographies on hMSC proliferation and differentiation^14^. The use of mathematical algorithms to generate feature shapes allowed us to screen cells in an unbiased and random manner allowing the discovery of novel or unexpected effects arising from cell-topographical interactions. Meanwhile, using a DNA spotter, Mei *et al.* fabricated an array of 576 unique polymeric materials that they examined for the clonal expansion of pluripotent stem cells (PSCs)^15^. By using high-throughput characterisation methodologies, they were able to systematically examine and develop structure-function relationships between material properties and biological performance. Using this approach they demonstrated that substrates with high acrylate content and a moderate wettability are optimal for PSC colony formation.

It should be noted, however, that the majority of studies to date have primarily focussed on screening biomaterials in 2D whereas most clinical applications require 3D scaffolds. Some initial studies have taken place which involved the generation of 3D salt-leached scaffolds^16^,^17^. Using two different polymer solutions pumped through a two-syringe pump, Chatterjee *et al.*^16^ were able to generate an array of scaffolds by changing the pumping rates of the different solutions. This method created an array of sponge scaffolds composed of two polymers that were then screened with osteoblasts. However, such a dual pumping method is restricted to screening just two materials. The same group has also initiated comparative studies between scaffolds of different strut dimensions, showing that cells are highly sensitive to scaffold structure with nanofibrous scaffolds able to drive hMSCs down an osteogenic lineage^18^.

In this study, we aimed to make use of electrospinning to generate a library of nanofibrous collagen-polymer hybrid scaffolds that were then screened with a murine ATDC5 chondrogenic cell line. We used poly(lactic acid) (PLA) and poly (lactic-co-glycolic) acid (PLGA) with different monomer ratios to blend with collagen which resulted in a number of unique chemical and mechanical environments. In this way, we were able to assess the effect of the changing chemical and mechanical environment of nanofibrous scaffolds on chondrocyte behaviour. ATDC5 cells have been shown to express the full cascade of events described for differentiation of epiphyseal chondrocytes^19^. Differentiation markers at the gene and protein level were evaluated and the surface and mechanical properties of the scaffolds were characterized with a view to developing structure-function relationships between the cell-scaffold constructs.

## Results

### Scaffold preparation

**Figure 1:**
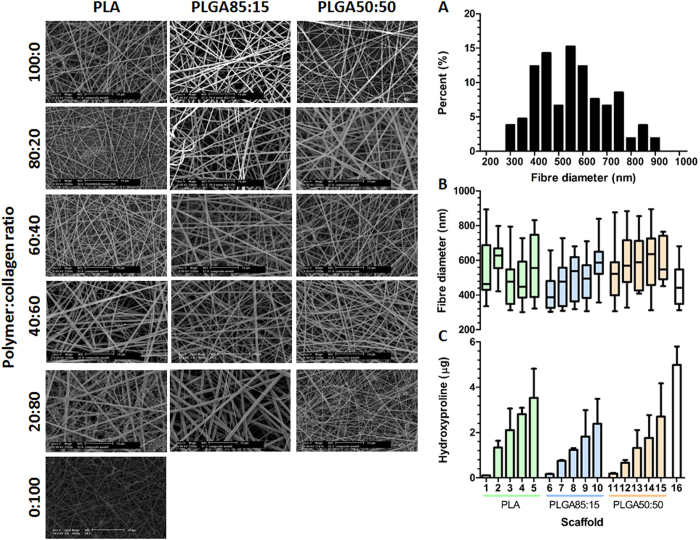
Generation of electrospun scaffold library. SEM images of the Polymer:collagen hybrid scaffolds displaying a similar morphology and fibre diameter. (**A**) Representative histogram of fibre diameters, generated for PLA scaffold, showing a normal distribution and a mean fibre diameter of 538 ± 107 nm. (**B**) Mean fibre diameters of all 16 scaffolds showing no significant differences in diameter (ANOVA, p = 0.6724 nm, N = 6). (**C**) Quantification of scaffold collagen content confirming the chemical composition of the hybrid scaffolds. The scaffolds 1–16 (**B**,**C**) correspond to the scaffold composition listed in Table 1.

All 16 scaffold compositions listed in Table 1 were successfully electrospun into fibrous meshes (Fig. 1). By adjusting the electrospinning parameters (Table 1), structural features of the scaffolds were kept constant throughout the array negating any influence that variable fibre diameter may have had on biological performance. A histogram of fibre diameters (Fig. 1A) displayed a normal distribution with a mean diameter of 538 ± 107 nm and no significant difference (p = 0.6724) detected between the fibre diameters of the 16 scaffolds (Fig. 1B). To evaluate the chemical composition of the hybrid scaffolds, the hydroxyproline assay was used to determine the collagen content of each scaffold (Fig. 1C). The hydroxyproline assay confirms that with each incremental rise of collagen in the electrospinning solution, the corresponding scaffolds displayed an equivalent increase in hydroxyproline content.

### Scaffold screening

**Figure 2:**
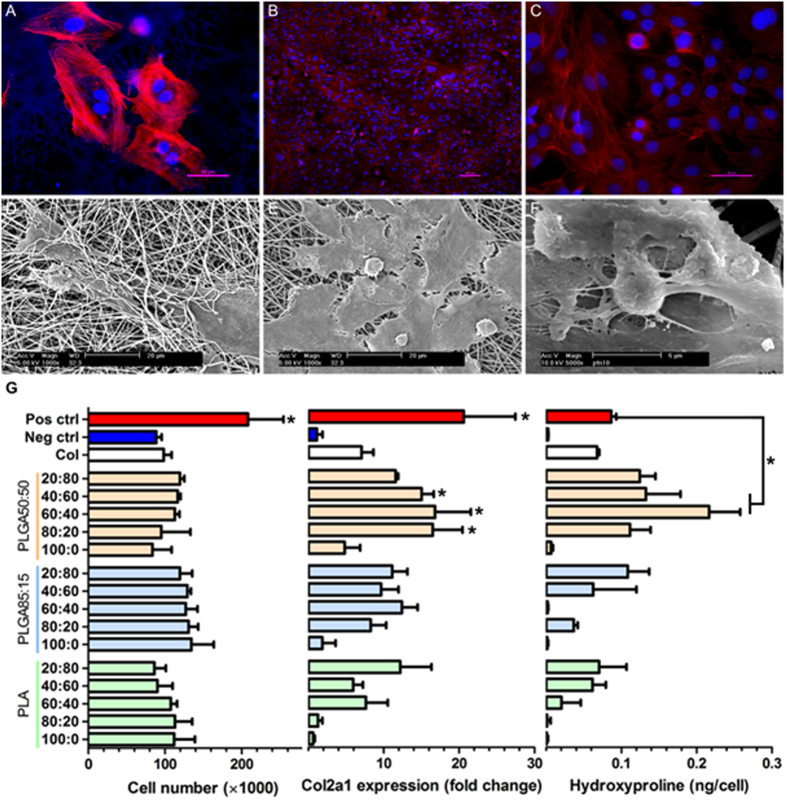
Representative fluorescent images with actin filaments in red and nucleas blue (A–C) and SEM (D–F) images of ATDC5 chondrocyted cultured on electrospun scaffolds 24 hours (A,D) and 14 days (B,C,E,F) after seeding. (**E**,**F**) provide a high magnification image of chondrocytes on the scaffold. Screening of electrospun scaffolds on day 14 (**G**). No significant differences in cell number detected between the scaffolds (ANOVA, p = 0.0581, N = 3) however the positive control is significantly higher than electrospun conditions (*p < 0.0001). Col2a1 gene expression of the cells on the scaffolds was compared to the positive control. There was no significant difference between col2a1 expression of the positive control and 3 of the scaffolds (marked with an *) which are all significantly higher than the remaining substrates (Dunnett’s test, p < 0.0001, N = 3). Similarly, hydroxyproline production on scaffolds was compared to the positive control using Dunnett’s multiple comparison test. Hydroxyproline production is at its maximum on five scaffolds which are higher than the positive control, however two are significantly so (marked *p < 0.0001, N = 3).

ATDC5 chondrogenic cell line was used for screening. Upon initial seeding, it can be seen that the cells adhered to the scaffold and were highly spread in nature (Fig. 2A) with some infiltration of the fibrous mesh taking place (Fig. 2D). Over time, as the cells proliferate and increase in number, they began to form a dense monolayer on the surface of the scaffold and were uniformly distributed (Fig. 2B,C). From the high magnification SEM images (Fig. 2E,F), in amongst the layer of highly spread cells, occasional spherical chondrocytes can be distinguished. There appeared to be protrusions emanating from the cell body and a dense layer of ECM was deposited on the surface of the scaffold.

In an effort to identify the optimal time point for screening the library of scaffolds, preliminary experiments were conducted whereby the ATDC5 cells were cultured on standard tissue culture well plates for a period of 4 weeks in both basic media (negative control) and differentiation media with the addition of ITS (positive control). Cell number, hydroxyproline content and collagen type II (Col2a1) gene expression were evaluated every week (Fig. S1). The Z-factor for each assay was then calculated with a value between 0 and 0.5 indicating an acceptable assay whereas a value between 0.5 to 1 suggesting an excellent assay. A negative value suggested an unacceptable assay. The earliest time point with a Z-factor between 0 and 0.5 was chosen as the optimal time point for screening, which was found to be at day 14.

The scaffolds were screened in basic media with no chemical additives and the same three assays – cell number, hydroxyproline and col2a1 expression were used as the biological end points (Fig. 2G). The scaffolds were compared to the negative control (TCPS) to evaluate the influence of the culturing substrate. The positive control (differentiation media with ITS) was used to check the putative response of the cells rather than a benchmark. No significant differences were noted in cell number between the scaffolds. However, the positive control was higher (p < 0.0001) indicating that the substrate the cells are cultured on does not affect proliferation, but the addition of ITS to the media has a significant influence. The substrates did have a significant influence over Col2a1 gene expression – a marker for chondrogenic differentiation. Expression levels were normalized to the negative control and were found to be highly variable on the scaffolds ranging from 0.64 to nearly 17 fold increase (Fig. 2G). Of the 16 scaffolds, three (composed of PLGA50:50 with 20, 40 and 60% collagen) were comparable to the positive control, with no significant differences detected whilst the remaining 13 displayed significantly reduced levels of col2a1 expression. Similarly, hydroxyproline production varied significantly on the substrates. Seven of the scaffolds had significantly lower levels of hydroxyproline compared to the positive control and a further seven were comparable to the positive control. Two scaffolds induced a significantly higher expression of hydroxyproline than the positive control and were selected for further examination. Both were composed of the synthetic polymer PLGA50:50 with 40 or 60% collagen referred to as P:C_6:4_ and P:C_4:6_ respectively.

### “Hit” validation

**Figure 3:**
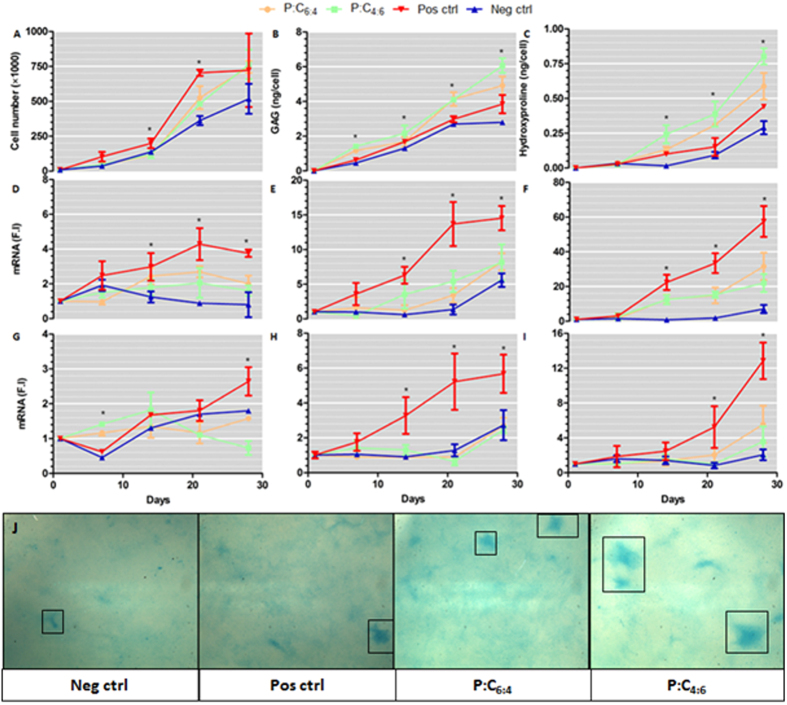
Validation of “hit” scaffolds identified from library screen. ATDC5 cells were cultured on P:C_6:4_ and P:C_4:6_ scaffolds as well as on standard tissue culture polystyrene in basic media (negative control) and with differentiation media (positive control). Cell proliferation was evaluated through measuring the levels of DNA present (**A**). sGAG (**B**) and hydroxproline (**C**) production per cell was quantified to determine the degree of chondrogenesis. Relative mRNA levels, expressed in fold induction (F.I) compared to the negative control at day 0, of Sox9 (**D**), aggrecan (**E**), col2a1 (**F**), RunX2 (G), col1a1 (**H**) and col10a1 (**F**) were examined with real-time qPCR (ANOVA, p < 0.0001, N = 6). Alcian blue staining of substrates confirms the increased presence of sGAG’s on the scaffolds (**J**). The boxes mark out nodules of sGAG deposition.

In an effort to validate the initial screen, time series experiments were conducted on the two “hit” scaffolds looking at cell proliferation, ECM production and chondrogenic gene expression (Fig. 3) over a four week period. Proliferation (Fig. 3A) was at its highest in the positive control, which by day 14 was significantly higher than the remaining 3 conditions, reaching a saturation point at about day 21 whereas cells on the remaining samples continued to proliferate. By day 28, no significant differences were detected between the samples.

To quantify the degree of chondrogenesis, sulphated GAG (sGAG) and total collagen content of each group was determined. Cells on both the scaffolds produced significantly more ECM than either the positive or negative controls cultured on tissue culture plastic. Statistically significant differences in sGAG levels were detectable by day 7 and were further amplified over the remaining culture period as the rate of sGAG expression was higher on the scaffolds. No significant differences were detected between the scaffolds. Alcian blue staining confirmed the increased presence of sGAG on the scaffolds (Fig. 3J). Similarly, significantly higher levels of collagen were detected on the scaffolds from day 14 onwards; by day 28 collagen levels were at their greatest on P:C_4:6_ which was slightly higher than P:C_6:4_.

To determine chondrocyte phenotype, gene expression levels were quantified (Fig. 3D–I). Expression levels for markers of chondrogenic differentiation (Sox9, aggrecan and col2a1) displayed broadly similar behaviour: the positive control induced the highest level of expression, followed by the scaffolds which were higher than the negative control. RunX2 and collagen type X are both indicative of hypertrophic differentiation. Whilst RunX2 levels are nominal in the four groups, by day 28 there is a slight spike on the positive control which also displays significantly upregulated levels of type X collagen. The positive control also exhibited increased levels of type I collagen which suggests a degree of de-differentiation. These results would suggest that there is a significant degree of phenotypic heterogeneity in the positive control which is not present when the cells are cultured on the scaffolds.

### Structure-function relationships

Physical properties of the scaffolds were measured with a view to developing relationships between scaffold properties and biological performance. Scaffold elastic modulus and adhesion forces were quantified with AFM whilst the wettability was determined through water contact angle (WCA) measurements. Whereas adhesion force did not correlate with the biological parameters measured (Fig. S2), a strong association between biological performance and the elastic modulus and wettability was detected.

#### Elastic modulus

**Figure 4:**
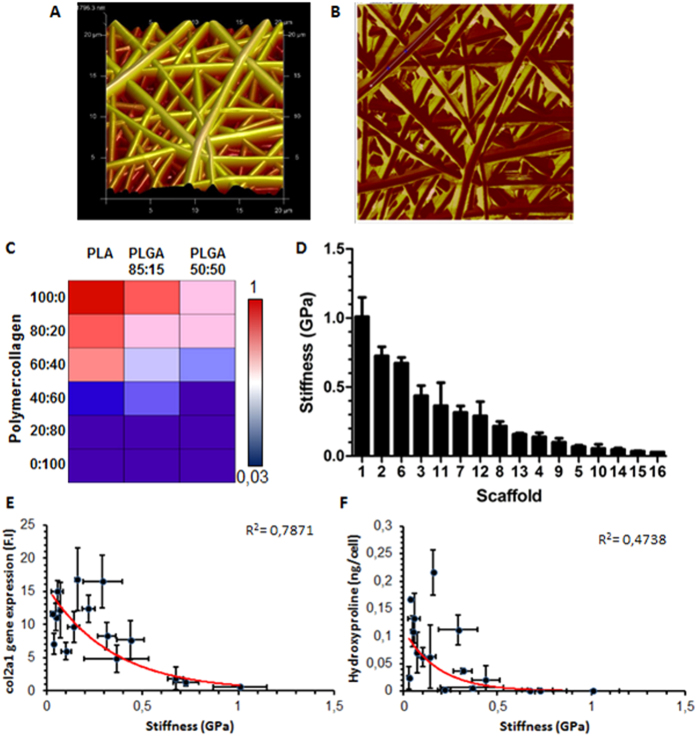
AFM analysis of scaffolds. Representative surface image of PLA scaffold (**A**). PeakForce scan of scaffold measuring DMT modulus of fibres (**B**). Mode average elastic modulus of scaffolds decreasing with the addition of collagen and increasing glycolide content (**C**). Linear range of elastic moduli of scaffolds ranging from the stiffest to the weakest (**D**). As stiffness increases, both col2a1 expression and hydroxyproline content decrease. A negative exponential fit results in a reasonable correlation between stiffness and col2a1 expression (R^2 ^= 0.7871) (**E**) and hydroxyproline (R^2 ^= 0.4738) (**F**).

Nanoindentation measurements via AFM allowed the characterization of the surface stiffness of the scaffolds. Surface images of the scaffolds were initially taken which corroborated the fibre morphology measurements, namely fibre diameter, made previously through SEM (Fig. 4A). PeakForce QNM measurements were taken, which provides a map of the DMT elastic modulus of the scaffolds (Fig. 4B). The mode average stiffness values were obtained for the 16 scaffolds which show a reduction in stiffness both with the addition of collagen and with an increase in glycolic acid (Fig. 4C). Values were obtained ranging from 30 MPa for collagen to 1.1 GPa for PLA (Fig. 4D). Col2a1 gene expression levels were found to decrease with increasing scaffold stiffness with an exponential regression analysis yielding an R^2^ value of 0.79 (Fig. 4E). Meanwhile, there was a weaker correlation between stiffness and hydroxyproline content on each scaffold with non-linear regression analysis resulting in an R^2^ value of 0.4738 (Fig. 4F). A closer examination of the cellular morphology on the stiff and soft scaffolds indicates the increased presence of active stress fibres on the stiff substrates which are not present on the softer scaffolds (Fig. S3).

#### Water contact angle

**Figure 5:**
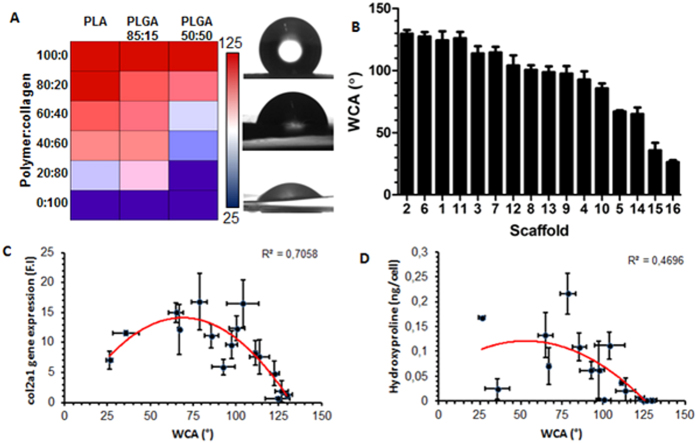
Water contact angle (WCA) measurements of electrospun hybrid scaffolds becoming more hydrophilic with the addition of collagen (A). Alongside the heatmap are representative images of the liquid drop corresponding to a highly hydrophobic (top), intermediate (middle) and hydrophilic (bottom) scaffold. Linear range of scaffold WCA measurement arranged from the most hydrophobic to hydrophilic (**B**). Non-linear regression analysis (2^nd^ order polynomial) on WCA versus col2a1 expression (R^2 ^= 0.7058) suggesting that an optimal wettability exists at approximately 75° (**C**). Hydroxyproline content plotted against WCA yields similar results with a poorer coefficient of determination (R^2 ^= 0.4696) (**D**).

Hydrophilicity-hydrophobicity of materials has previously been suggested as a key regulator of protein and cell adhesion. Water contact angle (WCA) was used to quantify the surface wettability of the scaffolds (Fig. 5A). It is evident from the heatmap that the addition of collagen resulted in a more hydrophilic surface and that scaffolds with a higher lactic acid content are more hydrophobic. Overall, the scaffolds exhibited a large range of WCAs – from 25.26° for collagen to 124.33° for PLA (Fig. 5B). WCAs were plotted against biological properties (col2a1 gene expression and hydroxyproline production) and regression analysis was carried out. A second-order polynomial fit returned an R^2^ value of 0.71 for col2a1 expression plotted against WCA (Fig. 5C) suggesting an optimal WCA for gene expression at approximately 75°. The correlation wasn’t as strong for hydroxyproline where R^2^ was reduced to 0.47 (Fig. 5D).

## Discussion

The versatile and inexpensive nature of electrospinning renders it a useful technique for the creation of micro- and nano-sized fibres. With the native ECM being composed of a dense fibrous network in a similar length-scale, electrospinning has been proposed as a promising technology for the creation of scaffolds for tissue engineering^20^. Electrospun scaffolds have particularly found favour amongst researchers for engineering cartilage^21^,^22^. Whilst numerous studies detail the use of electrospun scaffolds in cartilage engineering, there appears to be little consensus on the optimum properties required. In this study, we created a library of scaffolds by changing the chemical composition in a step-wise manner, to investigate the effects of scaffold properties on chondrocyte growth and differentiation.

In the native tissue, chondrocytes reside in a matrix rich in collagen, elastin and proteoglycans^23^. However, pure electrospun collagen does not exhibit the same unique mechanical properties as native collagen due to the compromising of the hierarchical structure upon hydration in the electrospinning process. Therefore, blending synthetic polymers with collagen allows the tailoring of the mechanical and bioactive properties of the scaffold. Poly (α-hydroxy esters), which includes poly (glycolic acid) (PGA), PLA and their copolymers PLGA are the most frequently used synthetic polymers in tissue engineering due their well characterised biodegradation behaviour and the fact that they are approved by regulatory bodies for clinical use. However, PGA exhibits an elevated degree of crystallinity limiting its solubility and resulting in a highly brittle material; therefore it was excluded from this study whereas PLA and the two copolymers PLGA85:15 and PLGA50:50 were used for blending with collagen.

With an additional methyl group, PLA based scaffolds were found to be more hydrophobic meanwhile the amorphous random copolymers PLGA85:15 and PLGA50:50 resulted in scaffolds with reduced elastic moduli. The addition of collagen had a dual influence of making the scaffolds more hydrophilic and further compromising the mechanical properties. The electrospinning parameters required to produce uniform fibres were broadly similar for all the scaffolds with mild adjustments made to flow rate and voltage to negate the formation of beads which are a common by-product of electrospinning. In previous studies of electrospinning collagen blends, a heterogeneous fibre population with webbing and ribbons was reported when high levels of collagen were electropsun^24^. These artefacts were not observed in the present study where the fibres were relatively homogenous in nature with fibre diameters uniformly distributed and relatively consistent.

The scaffolds library was screened with the chondrogenic cell line ATDC5 with a view to understanding how scaffold properties can influence chondrocyte phenotype. Three principle measurements were recorded: cell proliferation and collagen expression at the gene and protein level. Whilst proliferation was consistent on all the scaffolds, the scaffold library was able to elicit a wide range of responses for col2a1 gene and hydroxyproline expression. Regression analysis was used to correlate scaffold properties with biological performance. Whilst correlation does not imply causation, it does allow you to identify possible relationships between material properties and cell behaviour facilitating the generation of more cogent hypotheses.

It was observed that increasing the elastic modulus of the scaffolds resulted in a reduction in col2a1 expression. It’s well established that both intrinsic and extrinsic mechanical cues can influence cell behaviour^25^,^26^,^27^. Whilst the precise mechanotransductive pathways that regulate these signals are still unknown, RhoA-mediated cytoskeletal contractility has established itself as a critical node in mechanosensing^28^,^29^,^30^. The small GTPase RhoA, and it’s downstream effector ROCK, are known to be activated in response to increased ECM stiffness. Overexpression of RhoA/ROCK has been shown to suppress the chondrogenic transcription factor Sox9, which directly regulates col2a1 expression; thus stiffer scaffolds could reduce col2a1 production via RhoA/ROCK mediated Sox9 suppression^31^,^32^. A closer inspection of cell morphology on the soft P:C_4:6_ and stiff PLA indicates that there is a significantly greater degree of actin stress fibre formation on the stiff scaffolds. The induction of stress fibres is one of the classical activities of RhoA, indicating that RhoA is activated on stiffer scaffolds compared to the soft^33^.

Meanwhile, there was a quadratic fit between col2a1 and WCA. WCA is known to mediate the initial adhesion of proteins which then coordinates subsequent biochemical performance^34^,^35^. It’s interesting to note that a moderate WCA of approximately 75° appears to be optimal. The ideal wettability of a biomaterial surface is a balancing act as proteins are unable to adhere to high energy, hydrophilic surfaces due to the strong repulsive solvation forces arising from strongly bound water^36^,^37^. Hydrophobic surfaces, on the other hand, are generally considered to be more protein adsorbent, but highly hydrophobic materials replace the interfacial surface water with the hydrophobic domains of the proteins causing them to denature^38^,^39^. An intermediate WCA can adsorb proteins at a sufficient density and adsorptive strength whilst maintaining the adhesion motif integrity to promote cell receptor engagement.

Scaffolds inducing high levels of collagen gene expression also exhibited increased collagen deposition at the protein level, resulting in similar trends when comparing hydroxyproline levels with material properties. However, the correlations were not as strong suggesting other factors may also be involved. This was particularly apparent when comparing the cells cultured on scaffolds with the positive control. The latter exhibited increased gene expression levels but protein deposition was significantly lower. This could possibly be a consequence of limitations in free space restricting the deposition of ECM proteins when the chondrocytes were cultured on 2D well plates compared to porous, 3D scaffolds suggesting factors such as porosity and degradation rates could be influential. Once in the extracellular space, procollagen molecules undergo polymerisation and fibril formation to an extent dictated by both the genetic type of collagen and its association with other matrix components^40^. It’s interesting to note that the highly biodegradable PLGA50:50 with 40–60% collagen induced the greatest ECM protein deposition.

Additional gene and protein time series analysis verified the chondrogenic potential of these two PLGA50:50 scaffolds, P:C_6:4_ and P:C_4:6_. Whilst chondrogenic markers (Sox9, col2a1 and aggrecan) were upregulated on the positive control compared to the scaffolds, there was also a significant increase in the expression levels of col1a1, RunX2 and col10a1. Col1a1 is a marker for dedifferentiation whilst RunX2 and col10a1 are indicative of hypertrophic chondrocytes suggesting a degree of phenotypic heterogeneity which was not seen on either of the scaffolds. The ability of the scaffolds to maintain a stable chondrocyte phenotype could be due to the greater ECM deposited. Both collagen and sGAGs were significantly higher on the scaffolds providing a more natural environment for the cells. This would particularly be the case at later time points where the relatively fast degradation profile of the scaffolds would result in more free space for the accumulation of endogenous ECM proteins contributing to the long-term maintenance of phenotype.

The ability to systematically examine the influence of multiple parameters on cell behaviour should offer a more mechanistic understanding of cell-scaffold interactions, facilitating the improved design of future tissue engineered products. In this study, we used a combinatorial approach to examine the role of WCA and stiffness of electrospun scaffolds on chondrocyte behaviour. Significant variations in gene expression and collagen production were found on the 16 scaffolds with an intermediate wettability composed of the highly biodegradable PLGA50:50 and collagen, in two ratios (60:40 and 40:60), were optimal for chondrogenesis and maintaining a stable phenotype. This study was limited to 16 scaffolds as a proof-of-principle for the combinatorial approach to screening scaffolds. To maximise its potential, a greater number of scaffolds should be fabricated which not only differ in terms of their chemical make-up but also covering factors such as pore size, porosity and fibre diameter allowing the assessment of a wider range of scaffold properties which have been shown to influence cell behaviour. To achieve this, a number of technical developments are needed. Streamlined scaffold fabrication techniques are required for the automated fabrication of 3D scaffolds in a systematic manner with some control over scaffold properties. Appropriate automated bioassays are needed for evaluating cell behaviour in 3D and data analysis packages for the efficient analysis of high-throughput data.

## Conclusions

In this study, 16 electrospun scaffolds were systematically examined for their influence on chondrogenesis. Correlative relationships were identified between the stiffness and wettability of the scaffolds and chondrogenic differentiation. Stiff scaffolds that are highly hydrophilic (WCA < 60) or hydrophobic (WCA > 75) appear to inhibit chondrogenesis. Whereas soft scaffolds with intermediate wettability (WCA = 60–75) appeared to support the growth of chondrocytes. Furthermore, cells grown on scaffolds minimised phenotypic heterogeneity compared to cells on standard tissue culture plastic which displayed increased levels of de-differentiation and hypertrophy. Whilst a chondrogenic cell line was used in this study to examine scaffold properties, a more clinically relevant cell source should be used in the future to determine the translational feasibility of any promising scaffolds.

## Materials and Methods

### Scaffold preparation

Synthetic polymers poly(lactic acid) (PLA) and two forms of poly(lactic-*co*-glycolic acid) (all from Purac Biomaterials) with varying monomer ratios (PLGA85:15 and PLGA50:50) were used in combination with bovine collagen type I (kindly provided by Kensey Nash) to create a library consisting of 16 scaffolds. The polymer and polymer-collagen solutions were prepared by dissolving in 1,1,1,3,3,3-Hexafluoro-2-propanol (BioSolve BV) overnight at a concentration of 5% (w/v). For collagen, the concentration was increased to 8% (w/v) and was also prepared in 1,1,1,3,3,3-Hexafluoro-2-propanol. The ratio of collagen:synthetic polymer was varied in 20% increments.

A custom electrospinning chamber with environmental control (25 °C, 30% humidity) was used to fabricate the scaffolds. The desired solutions were loaded into a syringe and placed in a syringe pump (KDS 100, KD Scientific). A metallic needle was attached to the syringe tip which acted as the spinneret and aluminium foil was used as a collector. Electrospinning parameters (Table 1) were adjusted so that fibre diameter remained relatively constant amongst the scaffolds.

### Scaffold characterization

#### Scanning electron microscopy

The morphology of the fibres was observed using scanning electron microscopy (SEM) (XL 30 ESEM-FEG, Philips/FEI) following gold sputter coating. A minimum of 5 images per sample were taken and a minimum of 20 fibres were measured per image to determine fibre diameters using an in-house macro developed for Image J (National Institutes of Health, Bethesda, MD, USA).

#### Atomic force microscopy

Scaffold stiffness and morphology was determined via atomic force microscopy (AFM) using a Bruker Bioscope Catalyst microscope with a Nanoscope V controller (Veeco, Instruments). Cantilever stiffness was first calibrated using the thermal fluctuation method and the sensitivity of the optical deflection system was calibrated by measuring the force-displacement of a glass slide. Thereafter, an aluminium coated, silicon AFM tip of 150 KHz resonance frequency and 4.5 N/m nominal spring constant was driven into the sample at a rate of 0.25 m/s and the sample deformation measured by piezo displacement. A minimum of 5 regions were measured for each sample. Instrument specific software (Nanoscope analysis) was used to determine the elastic modulus and adhesion force of each scaffold.

#### Contact angle measurements

Contact angle analysis was carried out using a goniometer (DSA100E, Kruss, Germany) equipped with a high speed framing video recording system with a CCD camera. A 3 μL Milli-Q water drop was placed on the surface of each sample, in air, at room temperature. The manufacturer drop shape analysis software was used to determine the air-water contact angles. A minimum of 5 droplets were examined for each sample.

### Cell culture

Screening was performed using the murine ATDC5 chondrogenic cell line (ATCC) cultured in DMEM/F12 media (Gibco), supplemented with 5% fetal bovine serum (Lonza) and 1% Penicillin/Streptomycin (Invitrogen). Positive controls were cultured in the same media with the 1% Insulin-Transferrin-Selenuim (ITS) (Invitrogen), a potent inducer of chondrogenic differentiation. Cells were kept at 37 °C and 5% CO_2_ with the media being refreshed three times per week and cells trypsinised when a confluency of 70–80% was reached.

Scaffold discs with diameter of 15 mm were cut and incubated in 70% ethanol for two hours, washed thrice with PBS for 1 minute each, transferred to a non-treated 24 well plate (Nunc) and incubated in cell culture medium overnight. After removal of the media, scaffolds were seeded with 10000 cells and returned to the incubator.

### Biological assays

#### Proliferation

Cell numbers on the scaffolds were determined using the CyQuant DNA assay (Molecular Probes, Oregon, USA) following Proteinase K digestion. At the designated time points, cell culture media was aspirated off and the cell-scaffold constructs were washed thrice with PBS and frozen at −80 °C until further processing. Samples were then digested at 56 °C in a Tris-EDTA buffered solution containing 1 mg/ml Proteinase K, 18.5 μg/ml pepstatin A and 1 μg/ml iodoacetamide (Sigma). DNA quantification was performed following manufacturer’s instructions using a sepctrofluorometer (Viktor3, Perkin Elmer).

#### Gene expression analysis

Cells were lysed using Trizol (Invitrogen) and after the addition of chloroform and centrifugation (15 mins, 12000 *g*, 4 °C), the aqueous phase containing the RNA was collected, precipitated using isopropyl alcohol, transferred to Nucleospin RNA columns (Macherey-Nagel), and processed as per manufacturer’s instructions. The quantity and quality of RNA was analysed using an ND100 spectrophotometer (Nanodrop technologies, USA). cDNA was synthesized from 500 ng of RNA, using the iScript reaction mix (BioRad) according to the manufacturer’s protocol. Quantitative PCR was performed on an iQ5 detection system (Bio-Rad) and fold induction was calculated using the Pfaffl method^41^. Primer sequences are provided in Table S1.

#### Collagen quantification

Hydroxyproline levels were used as a marker for collagen synthesis. Samples were washed three times in PBS and hydrolysed in 12M hydrochloric acid (Sigma) at 120 °C for 2–3 hours. Hydroxyproline assay (Biovision) was used as per manufacturer’s instructions. Hydroxyproline levels of the empty scaffolds were measured in parallel to the cell-scaffold constructs, thereby allowing the measurement of collagen synthesized by the cells alone. Hydroxyproline levels were further normalized by cell number to get a per cell measurement.

#### Glycosaminoglycan quantification

Samples were washed with PBS and digested with Proteinase K as previously stated for DNA quantification. Sulphated glycosaminoglycan (sGAG) content was determined spectrophotometrically with 9-dimethylmethylene blue chloride (DMMB) (Sigma) staining in PBE buffer (14.2 g/l Na_2_HPO_4_ and 3.72 g/l Na_2_EDTA, pH 6.5) with a microplate reader (Bio-rad) to achieve a per cell measurement. For Alcian blue staining, the cells were rinsed with PBS and fixed in 10% formalin for 15 mins. They were then stained with 0.1% Alcian blue 8GS in 0.1M aHCl overnight.

#### Cell morphology

Cells were visualized on the scaffolds via SEM and fluorescent microscopy. At the end of the culture time point, cell culture media was removed and the samples were washed three times with PBS and fixed in 4% paraformaldehyde. For SEM, the samples were rinsed with PBS, the samples were dehydrated via an ethanol gradient and then submerged in hexamethyldisilazane (Sigma). The samples were then sputter coated with gold for SEM observations.

For fluorescent imaging, the samples were washed with PBS, blocked using 5% bovine serum albumin for 1 hour, and permeabilised using 1% Triton X-100 for 15 minutes. Samples were then stained with phalloidin (Invitrogen, 1:50 dilution) for 30 minutes and DAPI (Invitrogen, 0.1 μg/ml) for 10 minutes before visualization under a fluorescent microscope (Nikon E600).

## Additional Information

**How to cite this article**: Ahmed, M. *et al.* A combinatorial approach towards the design of nanofibrous scaffolds for chondrogenesis. *Sci. Rep.* **5**, 14804; doi: 10.1038/srep14804 (2015).

## Supplementary Material

Supplementary Information

## Figures and Tables

**Table 1:** Electrospinning parameters used in the construction of the scaffold library.

Scaffold	Polymer	Collagen (%)	Electrospinning parameters		
			Flow rate(ml/h)	Voltage (kV)	Workingdistance (mm)
1	PLA	0	1.2	15	180
2		20	1.0	15	150
3		40	1.0	15	150
4		60	0.9	15	150
5		80	0.8	15	150
6	PLGA85:15	0	1	15	150
7		20	1	16	150
8		40	0.8	17	150
9		60	0.8	17	150
10		80	0.8	17	130
11	PLGA50:50	0	1	17	150
12		20	0.9	17	150
13		40	0.9	17	150
14		60	0.8	17	130
15		80	0.7	17	130
16	N/A	100	0.35	15	70
