# Supplementary material for: A combinatorial approach towards the design of nanofibrous scaffolds for chondrogenesis
Source: Sci Rep. 2015 Oct 7;5:14804. doi: 10.1038/srep14804 (PMC4595832; doi:10.1038/srep14804)
Supplement: Supplementary Information [file srep14804-s1.doc]

**A combinatorial approach towards the design of nanofibrous scaffolds for chondrogenesis**

Maqsood Ahmed^1^, Tiago André da Silva Ramos^1,3^, Febriyani Damanik^1^, Bach Quang Le^1^, Paul Wieringa^1^, Martin Bennink^2^, Clemens van Blitterswijk^1^, Jan de Boer^1^ and Lorenzo Moroni*^1^

**Supplementary data**

**Table S1**: List of primers used for qPCR

| **Gene** | **Sequence** | **Efficiency (%)** |
| --- | --- | --- |
| GAPDH | AGGTCGGTGTGAACGGATTTG | 98 |
|  | TGTAGACCATGTAGTTGAGGTCA |  |
| Col2a1 | CAAGGCCCCCGAGGTGACAAA | 97 |
|  | GGGGCCAGGGATTCCATTAGAGC |  |
| Col1a1 | GCATGGCCAAGAAGACATCC | 89 |
|  | CCTCGGGTTTCCACGTCTC |  |
| Col10a1 | TTTCTGCTGCTAATGTTCTTGACC | 83 |
|  | AATGCCTTGTTCTCCTCTTACTGG |  |
| Sox9 | CGGAACAGACTCACATCTCTCC | 94 |
|  | GCTTGCACGTCGGTTTTGG |  |
| RunX2 | AGAGTCAGATTACAGATCCCAGG | 87 |
|  | TGGCTCTTCTTACTGAGAGAGG |  |
| Aggrecan | AGAACCTTCGCTCCAATGACTC | 91 |
|  | AGGGTGTAGCGTGTGGAAATAG |  |

**
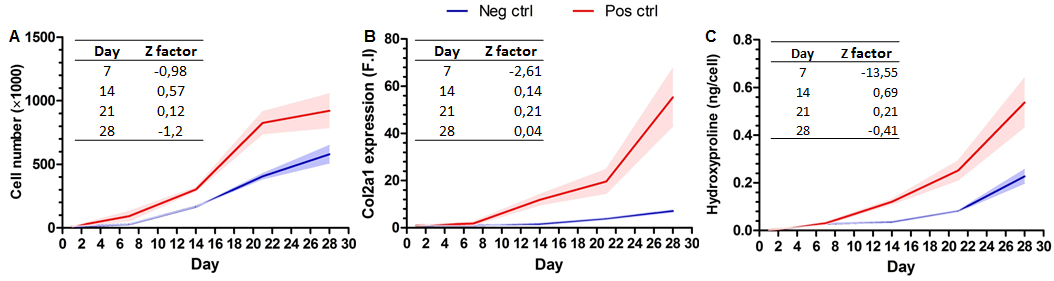
**

**Figure S1**: Proliferation (A), col2a1 expression (B) and hydroxyproline production (C) of the positive (red) and negative (blue) control samples over a 4 week period. The shaded area represents the standard deviation. The Z factor results are inset showing that at day 14, the Z factor is 0.57, 0.14, 0.69 for proliferation, col2a1 expression and hydroxyproline production respectively and is thus suitable to be used for screening purposes.


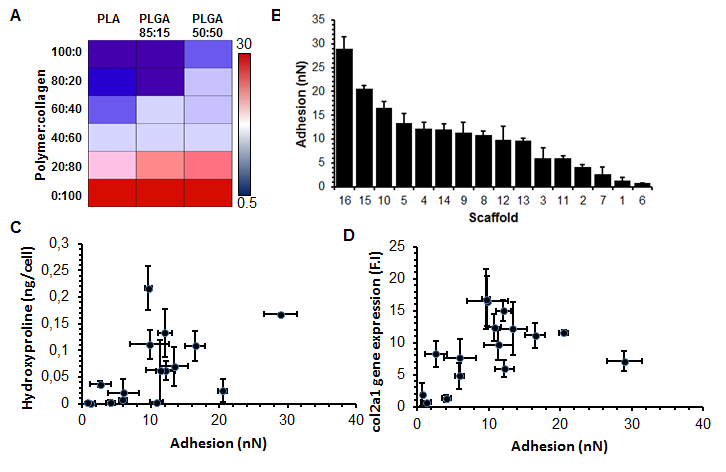


**Figure S2**: Adhesion measurements of scaffolds recorded from AFM (A). Linear range of scaffold adhesion arranged from highest to lowest (B). No correlation was detected between adhesion and either of the biological parameters (C and D)


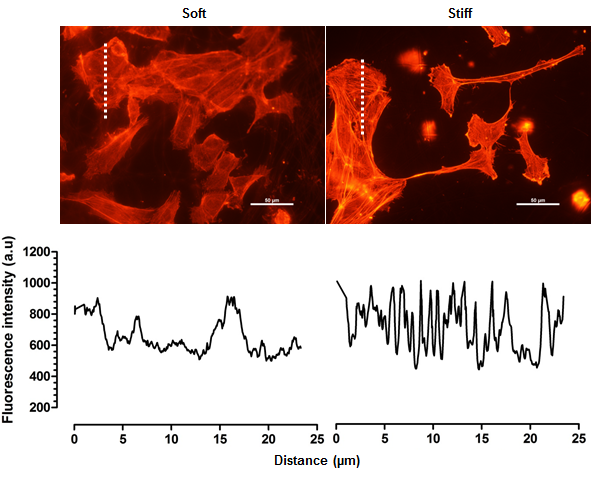


**Fig S3**: ATDC5 cells cultured on the soft P:C_4:6_ and the stiff PLA scaffold. The stiff substrate enhances stress fibre formation compared to the cells on the softer matrix. Fluorescence intensity is quantified across the line of the corresponding cells and confirms the minimal presence of stress fibres on the soft scaffold. On the stiff scaffold, a number of sharp peaks can be identified which correspond to the presence of actin stress fibres.
